# Dysregulated Cytokine Expression by CD4+ T cells from Post-Septic Mice Modulates both Th1 and Th2-Mediated Granulomatous Lung Inflammation

**DOI:** 10.1371/journal.pone.0020385

**Published:** 2011-05-31

**Authors:** William F. Carson, Toshihiro Ito, Matthew Schaller, Karen A. Cavassani, Stephen W. Chensue, Steven L. Kunkel

**Affiliations:** 1 Department of Pathology, University of Michigan Medical School, University of Michigan, Ann Arbor, Michigan, United States of America; 2 Department of Pathology and Laboratory Medicine, Veterans Affairs Ann Arbor, Healthcare System, Ann Arbor, Michigan, United States of America; Tulane University, United States of America

## Abstract

Previous epidemiological studies in humans and experimental studies in animals indicate that survivors of severe sepsis exhibit deficiencies in the activation and effector function of immune cells. In particular, CD4+ T lymphocytes can exhibit reduced proliferative capacity and improper cytokine responses following sepsis. To further investigate the cell-intrinsic defects of CD4+ T cells following sepsis, splenic CD4+ T cells from sham surgery and post-septic mice were transferred into lymphopenic mice. These recipient mice were then subjected to both TH1-(purified protein derivative) and TH2-(*Schistosoma mansoni* egg antigen) driven models of granulomatous lung inflammation. Post-septic CD4+ T cells mediated smaller TH1 and larger TH2 lung granulomas as compared to mice receiving CD4+ T cells from sham surgery donors. However, cytokine production by lymph node cells in antigen restimulation assays indicated increased pan-specific cytokine expression by post-septic CD4+ T cell recipient mice in both TH1 and TH2 granuloma models. These include increased production of T_H_2 cytokines in TH1 inflammation, and increased production of T_H_1 cytokines in TH2 inflammation. These results suggest that cell-intrinsic defects in CD4+ T cell effector function can have deleterious effects on inflammatory processes post-sepsis, due to a defect in the proper regulation of TH-specific cytokine expression.

## Introduction

Experimental evidence and epidemiological studies indicate that severe trauma, burn and shock can have a negative effect on subsequent immune responses. For example, survivors of severe sepsis exhibit decreased three- and five-year survival curves as compared to the healthy age-matched control population, and these survivors are at greater risk to develop opportunistic and nosocomial infections[1], [2]. Additionally, severe burn injuries can result in a suppression of subsequent pro-inflammatory responses, with deleterious effects on both the innate and adaptive immune system[3], [4]. Recent studies have also identified immunosuppression as a possible sequela of ischema/repurfusion injuries, such as stroke[5]. Therefore, studies aimed at dissecting the deficiencies in immune function following severe injury are essential for both the proper diagnosis and treatment of post-injury immunosuppression.

Animal models of severe sepsis are useful tools for dissecting the mechanisms underlying post-shock immunosuppression; for example, post-septic mice show increased susceptibility to solid tumor challenges[6], as well as opportunistic fungal[7] and bacterial[8] infections, as compared to control mice. This immunosuppression is manifested by numerous deficiencies in innate and adaptive immune cell function, which mimics the behavior of peripheral blood leukocytes in human patients post-sepsis. For example, dendritic cells from post-septic mice are deficient in their ability to produce IL-12 in response to TLR stimulus[9], and macrophages exhibit a decreased activation potential in response to LPS[10]. In addition, lymphocytes from post-septic mice and human patients exhibit numerous deficiencies in activation and effector function, including reduced proliferative capacity[11], [12], increased suppressive function[6], and dysregulated cytokine expression in response to T_H_1/T_H_2 cytokine stimulus[13]. Animal models of septic shock, including LPS injection and cecal ligation and puncture (CLP), can be used as model systems to dissect the cellular basis of post-septic immunosuppression[14].

Previous “two-hit” models of post-septic immunosuppression have focused on pathogens that are cleared mainly by the innate immune system (e.g. *Aspergillus fumigatus* airway challenge)[15]. Investigating the *in vivo* deficiencies in CD4+ T cell function in post-septic mice is problematic, as CD4+ T cells require interactions with antigen-presenting cells (such as DCs) for activation, and previous studies indicate that antigen-presenting cells suffer from their own activation deficiencies post-sepsis[16], [17], [18], [19]. Therefore, studies gauging the *in vivo* activity of post-septic CD4+ T cells would be clouded by the intrinsic defects in the antigen-presenting cell population in the post-septic animal. To effectively investigate the cell-intrinsic defects in post-septic CD4+ T cells in an *in vivo* model, the lymphopenic Rag2^−/−^ mouse was used as a recipient in an adoptive transfer model of granulomatous lung inflammation. In this system, splenic CD4+ T cells from mice subjected to sham surgery (control) or CLP (sepsis) are transferred into Rag2^−/−^ recipients, which are subsequently sensitized and challenged with model antigens in a bead model of granulomatous lung inflammation. In this model, any modulations in lung inflammation or immune responses *in vivo* can be ascribed to intrinsic deficiencies in the activation potential and effector function of the adoptively transferred donor CD4+ T cells, as myeloid cell functions in the recipient mice should be unaffected and comparable to control/healthy mice. The antigen-conjugated bead model of T_H_1/T_H_2 granulomatous lung inflammation was chosen as a secondary infection model as CD4+ T cells are required for granuloma formation[20], [21], and T cell-derived cytokines are essential for the quality of the cellular infiltrate[22].

Previous studies have suggested that inflammatory processes following sepsis are skewed in favor of T_H_2 responses and away from T_H_1[23]. Based on these studies, it was hypothesized that post-septic CD4+ T cells would show reduced T_H_1 effector function and increased T_H_2 effector function in the corresponding granuloma models. The results of this study indicate that *in vivo*, post-septic CD4+ T cells mediate increased T_H_2 and decreased T_H_1 granuloma formation, which correlates with previously published studies. However, cytokine production by lymph nodes of mice that received CLP CD4+ T cells indicated increased non-specific cytokine production in both T_H_1 and T_H_2 inflammation models, with no clear delineation between cytokine families in either model. These results indicate that cell-intrinsic defects in directed cytokine function in post-septic CD4+ T cells can modulate *in vivo* inflammation, directly affecting lung pathology. The relative inability of post-septic CD4+ T cells to produce directed T_H_1 or T_H_2 cytokines in response to antigen stimulus parallels *in vitro* reports, and further implicates the dysregulation of directed T_H_-type cytokine production by CD4+ T cells as one possible component of immune dysfunction post-sepsis.

## Results

### T_H_1 granuloma size was reduced in CLP RAG mice

**Figure 1:**
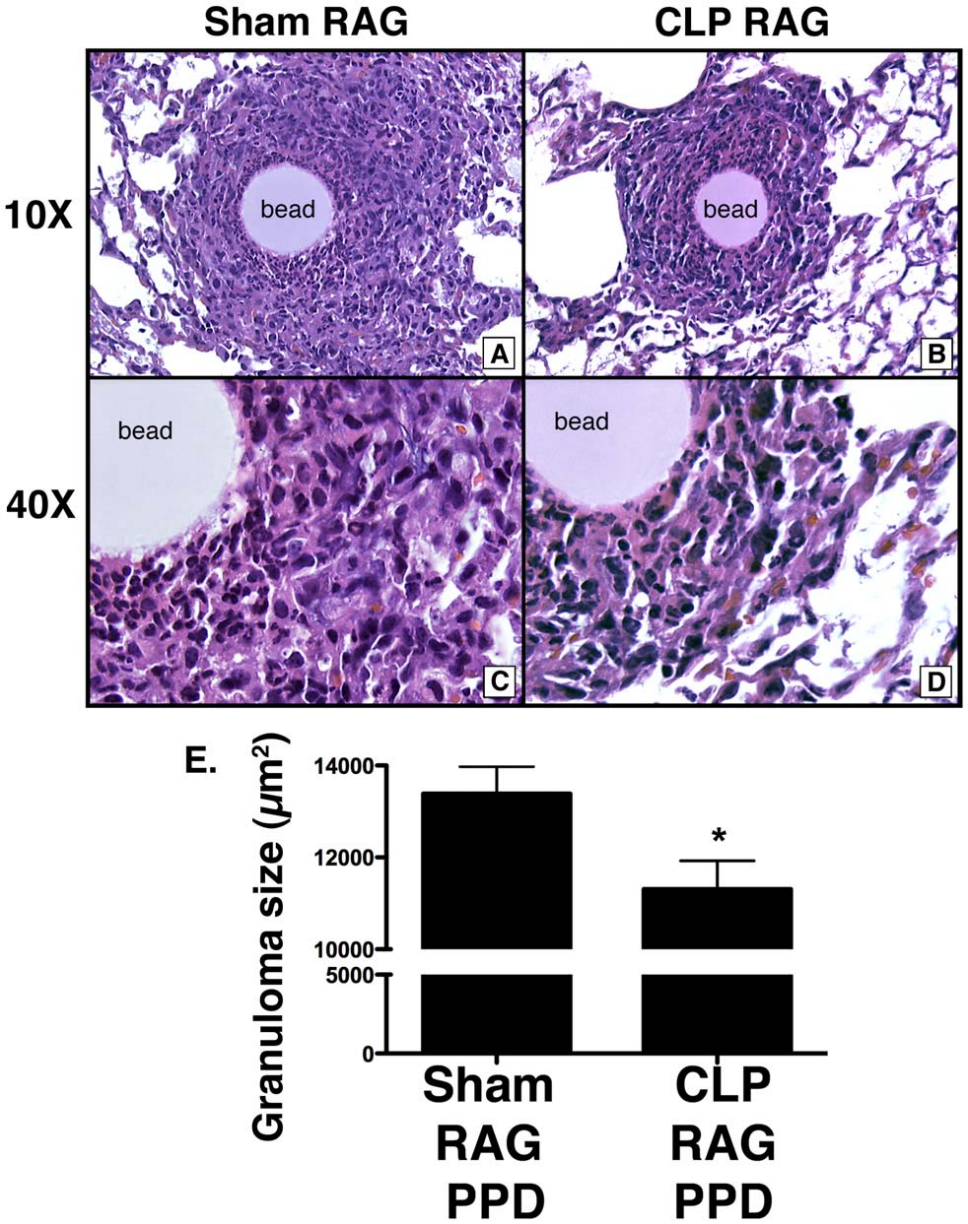
Decreased granuloma size in PPD-challenged CLP RAG lungs. RAG2^−/−^ mice that received i.v. injections of splenic CD4+ T cells from sham surgery or CLP donor mice were sensitized i.p. with PPD emulsified in CFA, and fourteen days following sensitization were i.v. challenged with PPD-coated sepharose beads. Four days following bead challenge, mice were sacrificed, and lung lobes were processed in a standard manner for histological analysis. H&E stains of sham RAG (A&C) and CLP RAG (B&D) lungs are shown at 10x (A&B) and 40x (C&D) magnification. “Bead” indicates the presence of the antigen-coated sepharose bead. (E) Average granuloma size in sham and CLP PPD-bead challenged lungs. Morphometric analysis of granulomas in H&E slides, 10 granulomas per lung, 5 lungs per group. Data presented is representative of two separate experiments, n = 5 per group. (*) = p<0.05 vs. sham RAG.

To assess the functional capacity of post-septic CD4+ T cells to mediate T_H_1-type granulomatous lung inflammation, RAG2^−/−^ mice that received CD4+ T cells from sham (Sham RAG) or CLP mice (CLP RAG) were subjected to the PPD sensitization and bead challenge model. Four days following intravenous (i.v.) injection of PPD-coated beads, mice were sacrificed and lung histology was analyzed to assess severity of inflammation. While both sham RAG (Fig. 1A) and CLP RAG (Fig. 1B) mice were able to generate granulomatous lung inflammation in response to PPD-bead challenge, the granulomas in the CLP RAG mice were of a smaller diameter than their Sham RAG counterparts (Fig. 1B). Total cellularity appeared reduced in CLP RAG mice, however the composition of the granulomas appeared similar between sham RAG (Fig. 1C) and CLP RAG (Fig. 1D) lungs, composed of mononuclear cells, lymphocytes and neutrophils. Repeated analysis of multiple lung slides confirmed the reduced size of CLP RAG granulomas, with a significant reduction in granuloma size observed across all lungs analyzed (Fig. 1E).

### Percentage and total number of CD4+ T cells were decreased in lungs of PPD-bead challenged CLP RAG mice

**Figure 2:**
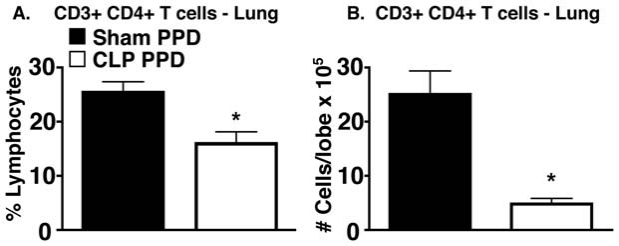
Percentage and total number of CD3+ CD4+ T cells in lungs of PPD-challenged mice. Lobes from sham and CLP RAG PPD-challenged mice were processed into a single-cell suspension and analyzed via flow cytometry for the presence of CD3+ CD4+ T cells. (A) Percentages of CD3+ CD4+ T cells were obtained by gating on the standard forward/side scatter profile of lymphocytes . (B) Total numbers of CD3+ CD4+ T cells were obtained by multiplying the percentages obtained via flow cytometry via the total viable cell count (hemacytometer & vital dye exclusion). Data presented is representative of two separate experiments, n = 5 per group. (*) = p<0.05 vs. sham RAG.

To determine if the reduction in granuloma size in CLP RAG mice was due to decreased numbers of adoptively transferred CD4+ T cells trafficking to the lung, flow cytometric analysis of lung tissue from sham and CLP RAG mice was performed. As RAG^−/−^ mice do not contain any endogenous mature CD3+ CD4+ T cells, it is expected that any mature CD3+ CD4+ T cells observed in peripheral tissues via flow cytometry will be donor-derived and not from the recipient mouse. The percentage of CD3+ CD4+ T cells (expressed as a percentage of total viable lung lymphocytes) was significantly reduced in CLP RAG lungs as compared to sham RAG mice at four days following i.v. PPD-bead challenge (Fig. 2A). Additionally, total numbers of CD3+ CD4+ T cells of CLP RAG mice were significantly decreased as compared to sham RAG mice (Fig. 2B), which also reflected a reduction in the total number of viable leukocytes recovered from the lungs of CLP RAG mice (per lobe: 9.70×10^6^±1.2 total cells for sham RAG, 3.86×10^6^ ±0.4 total cells for CLP RAG, p<0.01).

### Increased nonspecific cytokine expression in PPD- restimulated lymph node cultures from CLP RAG mice

To determine if the decrease in PPD-bead granuloma size in CLP RAG lungs was due to deficiencies in proinflammatory cytokine expression, protein expression in lung lobe digests and lymph node restimulation cultures were analyzed via multiplex bead assay for the presence of pro-inflammatory (T_H_1: IL-2, IFN-γ; T_H_2: IL-4, IL-5, IL-13; T_H_17: IL-17) and anti-inflammatory (IL-10) cytokines. For analysis of lung cytokines, single lobes from sham and CLP RAG mice were digested directly *ex vivo* for protein extraction; for lymph node analysis, single cell suspensions of total lymph node cells pooled from sham and CLP RAG mice were restimulated *in vitro* with PPD.

Analysis of lung protein from sham and CLP RAG mice showed no significant difference in the total amount of T_H_1, T_H_2 or T_H_17 cytokine production between sham and CLP RAG mice (Figure S1). Levels of IL-10 were significantly reduced in CLP RAG lungs as compared to sham (Figure S1D); however, the total levels of IL-10 in both sham and CLP RAG mice was relatively low compared to the levels of other pro-inflammatory cytokines. Surprisingly, while the size of the PPD granulomas in the lungs of CLP RAG mice were decreased as compared to sham RAG mice, there was no apparent effect on IFN-γ levels in total lung homogenate (Figure S1G).

**Figure 3:**
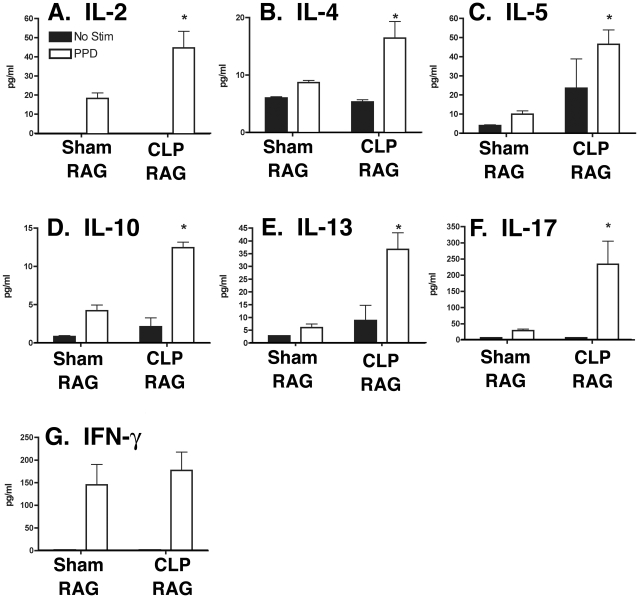
Cytokine expression in PPD-restimulated lymph node cultures from sham and CLP RAG mice. Single-cell suspensions of lymph nodes from PPD-sensitized sham and CLP RAG mice four days following PPD-bead challenge were restimulated with soluble PPD in cell culture for 48 hours, and cytokine expression in cell culture supernatants was analyzed via multiplex bead assay (Luminex). Data presented is representative of two separate experiments, with triplicate wells of samples from sham and CLP RAG mice, n = 5 mice per group. The limit of detection for each cytokine was routinely <5 pg/ml. (*) = p<0.05 vs. sham RAG, PPD-stimulated.

For analysis of antigen-dependent cytokine production by lymph node-resident lymphocytes, single cell suspensions of mediastinal lymph nodes from sham and CLP RAG mice were restimulated with PPD and supernatants were analyzed for the presence of various proinflammatory cytokines. In response to PPD stimulation, CLP RAG lymph node cultures produced significantly increased levels of the CD4+ T cell proliferative factor IL-2 as compared to sham RAG cultures (Fig. 3A). Surprisingly, T_H_2 and T_H_17 cytokine production by PPD-restimulated lymph node cultures from CLP RAG mice was increased as compared to sham RAG lymph node cultures (Fig. 3). CLP RAG lymph node restimulation assays exhibited PPD-specific increases in the T_H_2 cytokines IL-4 (Fig. 3B), IL-5 (Fig. 3C), and IL-13 (Fig. 3E), along with the T_H_2/immunosuppressive cytokine IL-10 (Fig. 3D) and the T_H_17 cytokine IL-17 (3F). In accordance with the lung homogenate results, levels of IFN-γ in PPD-restimulated lymph node cultures were equivalent between sham and CLP RAG mice (Fig. 3G).

Flow cytometric analysis of the lymphocytes present in sham and CLP lymph nodes was performed, as the cytokine production in lymph node cultures could be attributed to modulations in levels of CD4+ T cells present. Repeated analysis of individual lymph nodes from both sham (Figure S2A) and CLP RAG (Figure S2B) mice indicated no significant difference in the percentage or total number (1.89×10^4^±1.3 for sham LN, 1.58×10^4^±0.9 for CLP LN, p>0.05) of CD3+ CD4+ T cells.

### T_H_2 granuloma size was increased in CLP RAG mice

**Figure 4:**
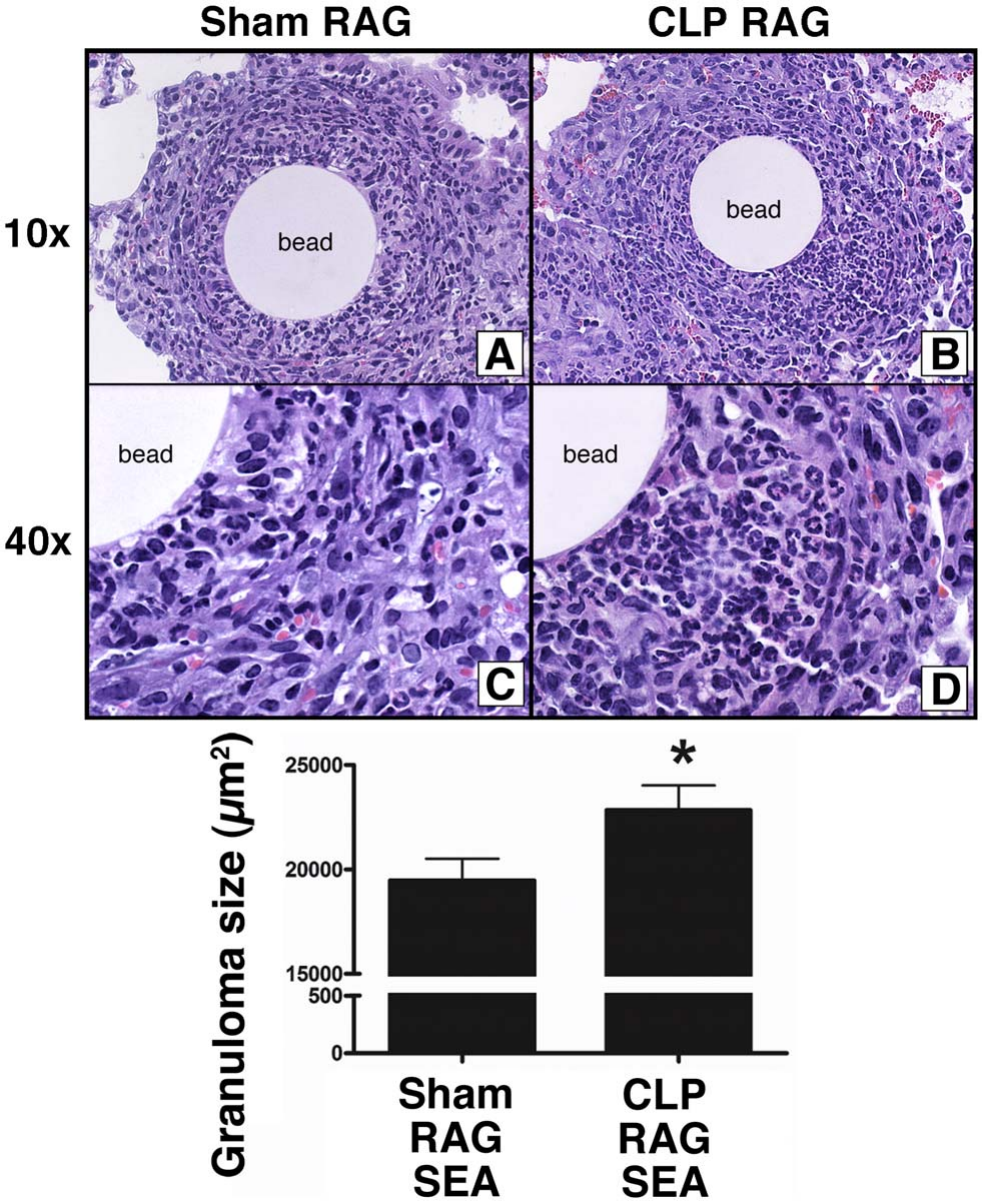
Increased granuloma size in SEA-challenged CLP RAG lungs. RAG2^−/−^ mice which received i.v. injections of splenic CD4+ T cells from sham surgery or CLP donor mice were sensitized i.p. with *Schistosoma mansoni* eggs, and fourteen days following sensitization were i.v. challenged with SEA-coated sepharose beads. Four days following bead challenge, mice were sacrificed, and lung lobes were processed in a standard manner for histological analysis. H&E stains of sham RAG (A&C) and CLP RAG (B&D) lungs are shown at 10x (A&B) and 40x (C&D) magnification. “Bead” indicates the presence of the antigen-coated sepharose bead. ce (E) Average granuloma size in sham and CLP SEA-bead challenged lungs. Morphometric analysis of granulomas in H&E slides, 10 granulomas per lung, 5 lungs per group. Data presented is representative of two separate experiments, n = 5 per group. (*) = p<0.05 vs. sham RAG.

To assess the functional capacity of post-septic CD4+ T cells to mediate T_H_2-type granulomatous lung inflammation, RAG2^−/−^ mice that received CD4+ T cells from sham (Sham RAG) or CLP mice (CLP RAG) were subjected to the SEA sensitization and bead challenge model. Four days following intravenous (i.v.) injection of SEA-coated beads, mice were sacrificed and lung histology was analyzed to assess severity of inflammation. While both sham RAG (Fig. 4A) and CLP RAG (Fig. 4B) mice were able to generate granulomatous lung inflammation in response to SEA-bead challenge, the granulomas in the CLP RAG mice were significantly increased as compared to sham RAG mice (Fig. 4B). This increase in granuloma size appeared to be largely due to an increase in the cellular infiltrate, with exaggerated levels of eosinophils in CLP RAG mice (Fig. 4D) as compared to sham RAG mice (Fig. 4C). Repeated analysis of multiple lung slides confirmed the increased size of CLP RAG granulomas, with a significant increase in granuloma size observed across all lungs analyzed (Fig. 4E).

### Percentage and total number of CD4+ T cells were equivalent in lungs of SEA-bead challenged sham and CLP RAG mice

**Figure 5:**
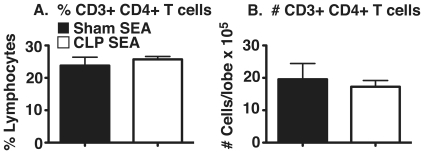
Percentage and total number of CD3+ CD4+ T cells in lungs of SEA-challenged mice. Lobes from sham and CLP RAG SEA-challenged mice were processed into a single-cell suspension and analyzed via flow cytometry for the presence of CD3+ CD4+ T cells. (A) Percentages of CD3+ CD4+ T cells were obtained by gating on total lymphocytes based on forward/side scatter profile. (B) Total numbers of CD3+ CD4+ T cells were obtained by multiplying the percentages obtained via flow cytometry via the total viable cell count (hemacytometer & vital dye exclusion). Data presented is representative of two separate experiments, n = 5 per group. (*) = p<0.05 vs. sham RAG.

To determine if the increase in granuloma size in CLP RAG mice was due to increased numbers of adoptively transferred CD4+ T cells trafficking to the lung, flow cytometric analysis of lung tissue from sham and CLP RAG mice was performed. The percentage of CD3+ CD4+ T cells (expressed as a percentage of total viable lung lymphocytes) was equivalent in CLP RAG lungs as compared to sham RAG mice at four days following i.v. SEA-bead challenge (Fig. 5A). In addition, total numbers of CD3+ CD4+ T cells were equivalent between sham and CLP RAG lungs, indicating that the apparent increase in SEA-granuloma size was not due to a local increase in infiltrating CD4+ T cells (Fig. 5B)

### Increased nonspecific cytokine expression in SEA- restimulated lymph node cultures from CLP RAG mice

To determine if the increase in SEA-bead granuloma size in CLP RAG lungs was due to increases in proinflammatory cytokine expression, protein expression in lung lobe digests and lymph node restimulation cultures were analyzed via multiplex bead assay for the presence of pro-inflammatory (T_H_1: IL-2, IFN-γ; T_H_2: IL-4, IL-5, IL-13; T_H_17: IL-17) and anti-inflammatory (IL-10) cytokines. For analysis of lung cytokines, single lobes from sham and CLP RAG mice were digested directly *ex vivo* for protein extraction; for lymph node analysis, single cell suspensions of total lymph node cells pooled from sham and CLP RAG mice were restimulated *in vitro* with SEA.

Analysis of lung protein from sham and CLP RAG SEA-bead challenged mice showed no significant difference in the total amount of T_H_1, T_H_2 or T_H_17 cytokine production between sham and CLP RAG mice (Figure S3). In contrast to the cytokine levels in RAG PPD-bead challenged mice, levels of IL-10 were similar in CLP RAG lungs as compared to sham (Figure S3D). However, in accordance to the results with IFN-γ in RAG PPD-bead challenged mice, there was no apparent difference in the levels of the T_H_2 cytokines IL-4 (Figure S3B), IL-5 (Figure S3C) or IL-13 (Figure S3E) in total lung homogenate between sham and CLP RAG SEA-bead challenged mice.

**Figure 6:**
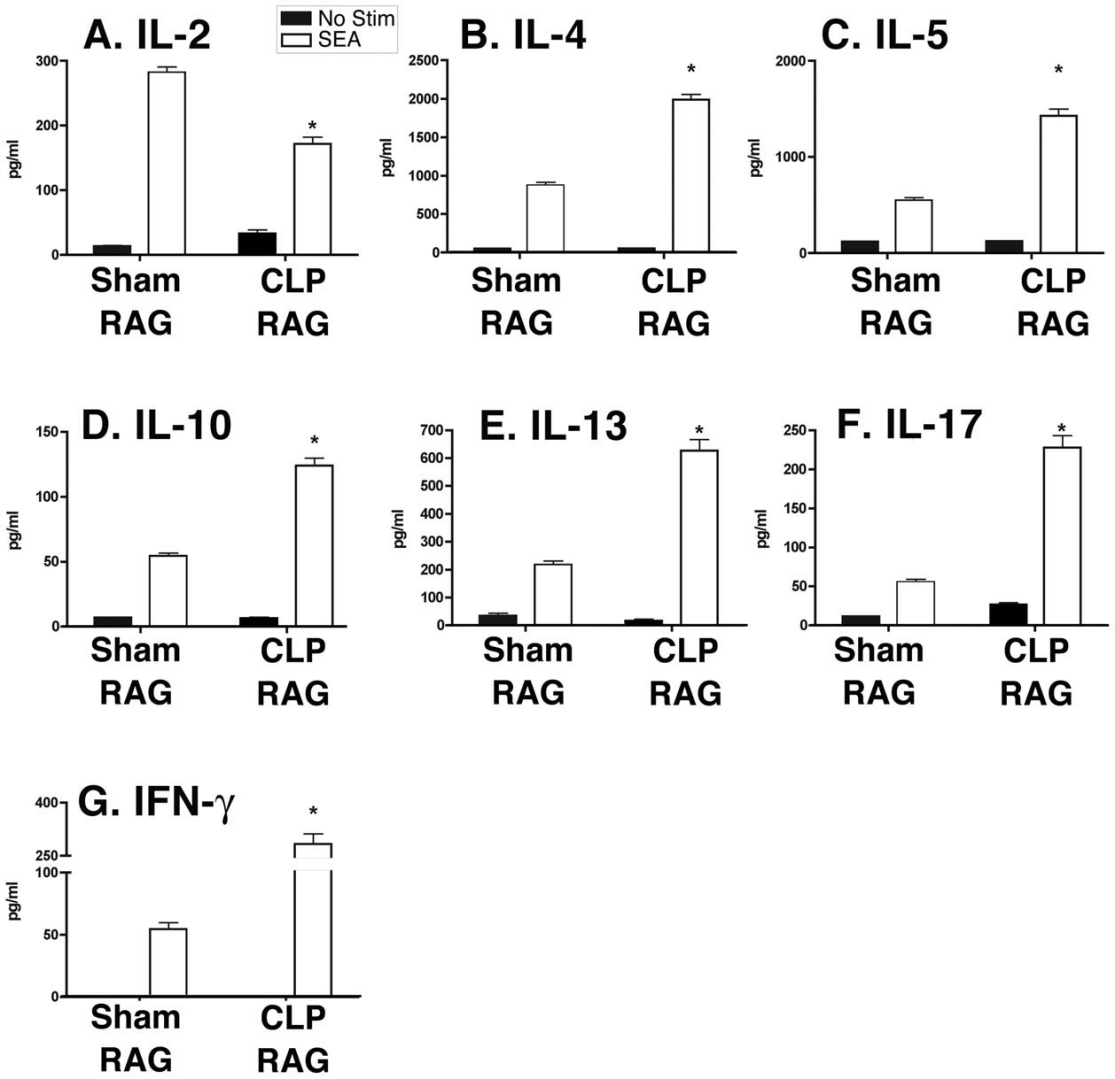
Cytokine expression in SEA-restimulated lymph node cultures from sham and CLP RAG mice. Single-cell suspensions of lymph nodes from *S. mansoni*-sensitized sham and CLP RAG mice four days following SEA-bead challenge were restimulated with soluble SEA in cell culture for 48 hours, and cytokine expression in cell culture supernatants was analyzed via multiplex bead assay (Luminex). Data presented is representative of two separate experiments, with triplicate wells of samples from sham and CLP RAG mice, n = 5 mice per group. The limit of detection for each cytokine was routinely <5 pg/ml. (*) = p<0.05 vs. sham RAG, SEA-stimulated.

For analysis of antigen-dependent cytokine production by lymph node-resident lymphocytes, single cell suspensions of mediastynal lymph nodes from sham and CLP RAG mice were restimulated with SEA and supernatants were analyzed for the presence of various proinflammatory cytokines. In response to SEA stimulation, CLP RAG lymph node cultures produced significantly decreased levels of the CD4+ T cell proliferative factor IL-2 as compared to sham RAG cultures (Fig. 6A). In accordance with the increased size of SEA-bead granulomas in CLP RAG mice, T_H_2 cytokine production by SEA-restimulated lymph node cultures from CLP RAG mice was increased as compared to sham RAG lymph node cultures (Fig. 3). CLP RAG lymph node restimulation assays exhibited SEA-specific increases in the T_H_2 cytokines IL-4 (Fig. 6B), IL-5 (Fig. 6C) and IL-13 (Fig. 6E), along with the T_H_2/immunosuppressive cytokine IL-10 (Fig. 6D). Surprisingly, CLP RAG lymph nodes also exhibited significantly elevated levels of the T_H_17 cytokine IL-17 (Fig. 6F), as well as levels of the T_H_1 cytokine IFN-γ (Fig. 6G). While sham RAG lymph node cultures also exhibited SEA-specific production of both IL-17 and IFN-γ, the levels of these cytokines were below the levels observed for T_H_2 cytokines from the same cultures. In a similar fashion as the RAG PPD lymph nodes, flow cytometric analysis indicated no significant difference in the percentage (Figures S2C and S2D) or total number (2.20×10^4^±1.1 for sham LN, 1.55×10^4^±0.9 for CLP LN, p>0:05) of CD3+ CD4+ T cells present in either sham or CLP RAG SEA lymph nodes, suggesting that the modulations in cytokine production by these lymph node cultures was not due to modulations in CD4+ T cells.

## Discussion

Previously published reports indicate that CD4+ T cells exhibit numerous functional deficiencies in response to the intense physiological stresses associated with sepsis, burn injury and trauma. In the case of severe sepsis, studies of both human patients and mouse models have identified numerous phenomena associated with post-septic CD4+ T cells; these include activation-induced cell death[11], [24], reduced proliferative capacity[12], [13] and modulations in pro-inflammatory gene expression[13], [25]. In particular, the generalized shift of post-septic immune responses away from T_H_1 and toward T_H_2 is often associated with deficiencies in post-septic CD4+ T cell function as well as accessory myeloid cells[26]. In many of these studies, the activation and effector function of post-septic CD4+ T cells was assayed either *in vitro* or in the context of post-septic antigen-presenting cells in the post-septic host animal *in vivo*. In the preceding study, cell-intrinsic defects in post-septic CD4+ T cell effector function were assayed *in vivo*, in the context of lung inflammation, using a lymphopenic mouse strain as a recipient for adoptive transfer. In this model, modulations in lung inflammation could be ascribed to post-septic T cells, as the antigen presenting cells in the recipient mice would not suffer from any post-septic immunosuppressive phenotypes.

Cytokine production by antigen-activated CD4+ T cells is central to the formation of granulomatous lung inflammation[21]. Depending on the qualitative nature of the infectious or experimental agent, the CD4+ T cells initiating the formulation of the granuloma can express either T_H_1 or T_H_2 cytokine profiles[22], [27]. In addition, the expression of T_H_1 and T_H_2 cytokines in the local lung environment is essential for the concurrent chemokine expression required for chemotaxis of accessory cells to the granuloma; in mouse models of T_H_1 and T_H_2 granuloma formation, blockade of the respective cytokine (IFN-γ or IL-4) results in significant down-regulation of chemokine mRNA in the lung[28]. Therefore, the cytokine profiles of the CD4+ T cells participating in the granulomatous response play an important role in dictating both the qualitative (size) and quantitative (cellularity) aspects of the lung lesion. Previous studies of T cell responses following experimental sepsis in mice (CLP) indicate that post-septic CD4+ T cells have modulated cytokine expression patterns *in vitro*, including increased *pan*-specific cytokine production directly *ex vivo* and non-specific T_H_ cytokine expression after skewing with recombinant cytokines[13]. In addition, putative regulatory T cells (CD4+CD25+) from the spleens of CLP animals exhibit increased suppressive function *in vitro*, and can suppress the production of IFN-γ from effector T cells in co-culture experiments[6]. The results of this study indicate that these post-septic cytokine imbalances appear to be present *in vivo*, and that these modulations in CD4+ T cell effector function are maintained in recipient animals that were not affected by post-septic immunosuppression.

Previous studies suggest that T_H_1-type responses are impaired in post-septic immunity. For example, *ex vivo* restimulation of leukocytes from post-septic patients indicates a decrease in the T_H_1-polarizing cytokine IL-12, essential for the development of T_H_1 CD4+ T cells[26]. Decreases in IL-12 production are also associated with post-septic susceptibility to lung infections with opportunistic fungi[29]. Production of IFN-γ by CD4+ T cells from post-septic patients and animal models is more controversial – certain studies report a decrease in IFN-γ production[30], [31] while others indicate no significant difference in the numbers of IFN-γ producing cells[26]. Previous studies of naïve (CD4+ CD62L+) T cells in CLP mice indicate increased IFN-γ production directly *ex vivo*, but decreased IFN-γ production following *in vitro* skewing to T_H_1[13]. In the present model, antigen restimulation of sham and CLP-recipient RAG mice indicates no significant difference in IFN-γ production, suggesting that the decreased granuloma size observed in the PPD-CFA T_H_1 granuloma model is not directly related to modulations in CD4+ T cell dependent IFN-γ production. However, CLP RAG lymph node restimulations indicate no significant difference in IFN-γ production in response to PPD; surprisingly, these cultures produced significantly increased amounts of both T_H_2 cytokines (IL-4, IL-5, IL-10 and IL-13), as well as the T_H_17 cytokine IL-17. Blockade of IL-4 in PPD-bead granuloma models in wild-type mice results in augmented IFN-γ production, and blockade of both IL-4 and IL-13 augments granuloma size[32]. Therefore, increased production of T_H_2-type cytokines in this PPD granuloma model may play a role in the decreased granuloma size observed in CLP RAG mice. The role of increased IL-17 in the CLP RAG PPD lymph nodes is less clear; previous studies indicate that IL-17-producing CD4+ T cells may promote the chemotaxis of IFN-γ -producing T cells to the granuloma[33], and loss of IL-17 disrupts granuloma formation in the lung in response to mycobacteria[34]. In this model, the maintenance of IFN-γ in the presence of increased T_H_2 cytokines may be due to an IFN-γ-promoting mechanism of increased IL-17. However, this explanation is purely speculative, and does not take into account the decreased granuloma size in the CLP RAG PPD lungs.

In addition to decreased T_H_1 responses, post-septic immunity is characterized by increased T_H_2-type responses. In post-septic patients, the ratio of IFN-γ to IL-4 producing peripheral blood CD4+ T cells appears skewed towards IL-4[23], and the maintenance of T_H_1 T cells following sepsis appears to be negatively affected by a unique susceptibility to apoptosis and activation-induced cell death[11], [35]. Following CLP, naïve CD4+ T cells exhibit a pan-specific cytokine response *ex vivo*, including increased IFN-γ and IL-4; however, production of IFN-γ is observed even after skewing to T_H_2 *in vitro* with exogenous cytokines[13]. In the present model, T_H_2 responses elicited in the CLP RAG SEA mice were indicative of both patient and animal model results. For example, the increased granuloma size in CLP RAG SEA lungs was consistent with the concept of increased T_H_2 responses by post-septic immune cells. In addition, the increase in IL-4, IL-5 and IL-13 production by CLP RAG SEA lymph nodes *in vitro* suggests one possible mechanism for the increased T_H_2-type lung lesion.

However, the concomitant increase in the T_H_1 cytokine IFN-γ and the T_H_17 cytokine IL-17 in the lymph node restimulations was surprising, as the increased presence of these T_H_2-opposing cytokines may be expected to interfere with the formation of the T_H_2 SEA granuloma. Interestingly, increases in IL-17 production appears to correlate with increased granuloma size in lesions mediated by *Schistosoma mansonii* eggs, both in the lung[36] and in the liver[37]. Therefore, the increases in IL-17 production mediated by the CLP CD4+ T cells could play a role in the increased size of the SEA-bead granulomas independent of increased T_H_2 cytokine production.

Despite being characterized as a T_H_2-type lesion, IFN-γ appears to play an important role in governing the size of *Schistosoma* and SEA-elicited granulomas. For example, mice deficient in either IFN-γ or the IFN-γ receptor show decreased granuloma size and associated immunopathology in liver models of egg challenge[38], [39]. These results suggests that in the opposite fashion, the increase in IFN-γ production in CLP RAG SEA lymph nodes may mediate increases in granuloma size rather than antagonizing the T_H_2 granuloma formation by suppressing immune responses via T_H_1-mediated cytokines. However, in contrast to the liver models, *in vivo* depletion of IFN-γ in the SEA-bead model results in an increase in granuloma size; this effect is observed when neutralizing antibodies are delivered at the time of bead challenge[27]. Based on these disparate reports in varying models of *Schistosoma*-antigen mediated granuloma formation, it appears that modulations in IFN-γ alone may not fully explain the increase in SEA granuloma size in CLP RAG lungs.

Surprisingly, despite the increase in IFN-γ production, levels of IL-2 were decreased in CLP RAG SEA lymph nodes. IL-2 is classically associated with T_H_1-type responses; however, recent studies indicate that IL-2 also plays an important role in stabilizing Th2 cytokine responses through regulating the accessibility of the *Il4* gene locus through chromatin remodeling[40], [41]. Therefore, the reduction in IL-2 production seen in the Th2-model lymph node restimulation assays may reflect a suppression of “Th2-type” IL-2 production, rather than evidence of decreased Th1-type responses. In addition, IL-17 production was increased in SEA-stimulated CLP RAG LN supernatants as compared to sham LN, suggesting an increased presence of Th17 T cells. As IL-2 is known to antagonize IL-17 production[42], the observed decrease in IL-2 may reflect this increased propensity towards Th17 cytokine responses.

One possible explanation for the modulation in lung granuloma size in both the PPD and SEA bead models is via a decreased trafficking of leukocytes to the lung following bead challenge. Our initial hypothesis is that any such deficiency in cell trafficking would be evidenced by deficiencies in CD4+ T cell migration to the lung, as these are the only cells that would theoretically differ in effector function in either the sham or CLP RAG mice. This may be an important phenomenon in the decreased granuloma size in the CLP RAG PPD lungs, as the percentage and total number of CD4+ T cells were decreased in these lungs as compared to sham RAG PPD. However, there was no concurrent increase in CD4+ T cell trafficking to the lungs of CLP RAG SEA lungs as would be expected, suggesting that modulations in CD4+ T cell trafficking may not be sufficient to explain the modulation in granuloma size in these models.

To further investigate the possibility that the modulation in granuloma size in both PPD- and SEA-bead challenged CLP RAG mice was due to modulated recruitment of leukocytes to the lung, analysis of chemokine expression in the lung was performed (Figure S4). Expression levels of CCL11 (Eotaxin), CCL22 (MDC) and CXCL10 (IP-10) were assessed via analysis of mRNA transcript levels. The indicated chemokines were chosen due to their relative importance for T_H_1 (ex. CXCL10) vs. T_H_2 (ex. CCL11) inflammation, as well as their previous implication in the sensitization and bead challenge model of granulomatous lung inflammation used in this study. However, modulations in lung chemokine mRNA did not correlate well with the CD4+ T cell trafficking data, as the only modulated transcript observed was CXCL10 (IP-10), which is often considered an important chemotactic factor for T_H_1 T cells[43]. The decreased trafficking of CD4+ T cells to the lungs of CLP RAG PPD mice despite increased CXCL10 production may indicate an inability of post-septic CD4+ T cells to respond to CXCR3-mediated signals. However, this conclusion is clouded by the concomitant decrease in CXCL10 mRNA in CLP RAG SEA lungs with no effect on CD4+ T cell trafficking to the lung. Combined with the equivalent CD4+ T cell composition of sham and CLP RAG lymph nodes in both the PPD and SEA models, the modulation in granuloma size does not appear to be due specifically to modulations in CD4+ T cell chemotaxis to the lungs or local draining lymph nodes.

Studies in both patients and animal modes indicate that post-septic immune dysfunction has a significant cellular component, with both innate and adaptive immune cells exhibiting deficiencies in activation and effector function. Previous studies investigating post-septic CD4+ T cell function have been limited by the requirement of accessory cells (such as antigen-presenting cells) for their activation; as a consequence, *in vivo* studies are affected by the presence of post-septic accessory cells that suffer from their own deficiencies. Moreover, *in vitro* studies of post-septic CD4+ T cell function can be used to assay cell-intrinsic defects in activation and effector function, but do not accurately assay the ability of the cells to mediate *in vivo* inflammation. In the present study, use of the RAG^−/−^ mouse as a recipient animal model for adoptive transfer allows for the assessment of post-septic CD4+ T cell function *in vivo* in the context of T_H_1 and T_H_2 inflammation, along with accessory cells that are wild-type in nature and not affected by post-septic deficiencies in function. The results of this study indicate that post-septic CD4+ T cells exhibit a general propensity for mediating T_H_2 inflammatory processes, as evidenced by decreased T_H_1 and increased T_H_2 granuloma size. Additionally, as seen with *in vitro* studies, post-septic CD4+ T cells exhibit a deficiency in their ability to properly commit to either the T_H_1 or T_H_2 inflammatory program, as both PPD and SEA-challenged lymph nodes from CLP T cell-recipient mice exhibit increased *pan*-specific cytokine production, including increases in T_H_17 cytokine. These results further highlight the cell-intrinsic defects in post-septic CD4+ T cell effector function, and suggest that the T_H_ bias observed in post-septic patients may not be entirely corrected by immunotherapy with wild-type antigen-presenting cells. The inability of post-septic CD4+ T cells to commit to either the T_H_1 or T_H_2 lineage may be significant for survivors of severe sepsis, especially in regards to secondary infections of the lung which require a directed cytokine response for clearance of the invading microorganism.

## Materials and Methods

### Ethics Statement/Mice

Female C57BL/6 and B6.129S6-*Rag2^tm1Fwa^* N12 (RAGN12, Rag2^−/−^) mice (6–8 wk of age; Taconic Farms, Germantown, NY, USA) were housed under specific pathogen-free conditions at the Unit for Laboratory Animal Medicine of the University of Michigan and treated in accordance with the guidelines of the animal ethical committee. All experiments were done with the approval of the University of Michigan Committee for Use and Care of Animals (UCUCA) under protocol 7689 (approval dates 02/27/2009–02/27/2012).

### Cecal Ligation and Puncture

CLP surgery was performed on mice as described previously[44]. For CLP, the cecum was punctured seven times with a 21-gauge needle. Both sham surgery and CLP mice were treated with the antibiotic INVANZ (Ertapenem, Merck & Co., Inc., Whitehouse Station, NJ) administrated at 75 mg/kg via intraperitoneal injection beginning at 6 hours after surgery and re-injected every 24 hours until day 3 after surgery. The average mortality rate for mice subjected to CLP in this study was 40–60% by day 4 after surgery.

### Adoptive transfer, sensitization and bead challenge model of granulomatous lung inflammation

Spleens from sham and CLP mice were harvested at day 14 post-surgery and processed in a standard manner. Briefly, spleens were mechanically dispersed into a single cell suspension using a 40 µm filter, and ammonium chloride lysis buffer was used to lyse erythrocytes. For purification of CD4+ T cells, ferromagnetic beads were utilized (CD4+ T cell isolation kit, Miltenyi Biotech, Auburn, CA) according to the manufacturer's instructions. Following purification, isolated cells were enumerated using a hemacytometer and trypan blue staining for the enumeration of viable cells. For transfer, 2.5×10^6^ CD4+ T cells from either sham or CLP donor mice were transferred via intravenous injection (tail vein) into recipient Rag2^−/−^ (RAGN12) mice (Figure S5). Three days (72 hr) following adoptive transfer, recipient Rag2^−/−^ mice were sensitized with either 20 µl of purified protein derivative (PPD; 1 mg/ml) emulsified in 250 µl Complete Freund's Adjuvant via subcutaneous injection for the generation of T_H_1 responses, or 3000 *Schistosoma mansoni* eggs in 300 µl of 1.7% NaCl solution via intraperitoneal injection for the generation of T_H_2 responses. Fourteen days following sensitization, mice were challenged via tail vein injection with 5500 Sepahrose 4B beads coated with either PPD (T_H_1) or soluble egg antigen (SEA) (T_H_2), suspended in 500 µl phosphate buffered saline. Four days following bead challenge, mice were sacrificed for analysis, as this represents the peak of lung lesion size in both T_H_1 and T_H_2 bead granuloma models[45]. For each experimental group, recipient mice were subjected to either T_H_1 or T_H_2 models within a single experiment, with 4–5 recipient mice per donor group receiving either sham or CLP-derived CD4+ T cells, respectively. For lung tissue analysis, two of the five lung lobes from each individual mouse were reserved for histological examination; the remaining three lobes were saved for RNA, protein and flow cytometry analysis, respectively.

### Histological examination

Individual excised lung lobes were inflated and fixed with 10% buffered formalin for morphometric analysis. The areas of the granulomas were measured in a blinded fashion on H&E-stained sections of paraffin-embedded lungs using computer-assisted morphometry as previously described[46]. Morphometric analysis was performed on an average of 10 granulomas per lung, with attention to the measurement of granulomas with similar sephadex bead diameters.

### Flow cytometry

Collagenase-digested (Sigma-Aldrich, St. Louis, MO) lung lobes and undigested lymph nodes from animals were processed into single cell suspensions by processing tissues through sterile 40-mm filters, and ammonium chloride lysis buffer was used to eliminate erythrocytes. Cells were stained with the following fluorescent antibodies in flow cytometry buffer (phosphate buffered saline, 1% w/v bovine serum albumin, 0.05% w/v sodium azide): Purified αCD16/32 (Fc Block) (2.4G2, BD Biosciences, San Jose, CA), FITC-αCD8a (53–6.7, BD Biosciences), FITC-αCD3ε (145-2C11, BD Biosciences), PeCy7-αCD45 (Ly5, eBioscience, San Diego, CA), PeCy7-αCD3ε (17A2, BioLegend, San Diego, CA), Pacific Blue- (RM4-5, BioLegend) or Pacific Orange (RM4-5, Invitrogen, Carlsbad, CA) -αCD4, and Pacific Blue-αCD45 (30-F11, BioLegend). Cells were fixed in 4% paraformaldehyde and analyzed on a LSR II (BD Biosciences). Flow cytometry data was analyzed using FlowJo 9.0.1 (Tree Star, Ashland, OR, USA).

### Multiplex cytokine analyisis

Concentrations of indicated cytokines in culture supernatants and lung homogenates were analyzed using a Luminex Bio-Plex 200 system (Bio-Rad, Hercules, CA, USA) according to the manufacturer's protocol, as previously described[9]. Cytokine levels in lung homogenates were normalized to the protein (in milligrams) present in cell-free preparations of each sample measured by the Bradford assay, as described previously[46]. Plates were washed and read using a Luminex Bio-Plex 200 system plate reader. For cytokine analysis, murine stock cytokines of known concentrations (provided with the kit) were used to generate standard curves. The threshold of each cytokine was routinely <5 pg/mL.

### Reverse transcription and real-time quantitative PCR analysis

Total RNA was isolated from whole lungs using TRIzol (Invitrogen) according to the manufacturer's instructions. In brief, a total of 1 µg of RNA was reverse transcribed to yield cDNA in a 25-µl reaction mixture containing 1× first strand buffer (Life Technologies; Invitrogen), 250 ng oligo (dT) primer, 1.6 mmol/l dNTPs (Invitrogen), 5 U RNase inhibitor (Invitrogen), and 100 U of Moloney murine leukemia virus reverse transcriptase (Invitrogen) at 38°C for 60 minutes. The reaction was stopped by incubating the cDNA at 94°C for 10 minutes. Real-time quantitative PCR analysis was performed by using the ABI 7700 Sequence Detector System (PE Applied Biosystems). Thermal cycling was performed at 50°C for 2 minutes and 95°C for 10 minutes, followed by 40 cycles of amplification at 95°C for 15 seconds and 55°C for 1.5 minutes for denaturing and annealing, respectively. Primer sets for the indicated transcripts were provided by the manufacturer (Applied Biosystems).

### Statistical Analysis

Significance was calculated utilizing repeated measures ANOVA when necessary, followed by *post hoc* Bonferroni tests for significance between experimental groups. For single-group analysis, two-tailed Student's *t*-tests were used to determine significance. In all cases, *p*<0.05 were considered statistically significant. Data analysis was performed with GraphPad Prism v5.0a for Macintosh (GraphPad software, San Diego, CA, USA).

## Supporting Information

Figure S1 **Cytokine expression in PPD-bead challenged lungs from sham and CLP RAG mice.** Total protein from lobes of lungs from sham and CLP RAG mice was isolated via mechanical dispersion, and clarified via centrifugation. Cytokine levels in lung protein were analyzed via multiplex bead assay (Luminex), and protein levels were standardized by the total protein in each lung sample (obtained via Bradford protein assay). Data presented is representative of two separate experiments, with triplicate wells of samples from sham and CLP RAG mice, n = 5 mice per group. The limit of detection for each cytokine was routinely <5 pg/ml. (*) = p<0.05 vs. sham RAG, SEA-stimulated.
(TIFF)

Figure S2 **Flow cytometric analysis of lymph nodes from sham and CLP RAG mice.** Individual lymph nodes from either sham (A&C) or CLP (B&D) PPD-challenged (A&B) or SEA-challenged (C&D) mice were processed individually and analyzed for the presence of CD3+ CD4+ T cells. Representative two-color plots were generated by gating on viable lymphocytes based on forward scatter (size) and side scatter (complexity) profiles, n = 4 mice per group.
(TIFF)

Figure S3 **Cytokine expression in SEA-bead challenged lungs from sham and CLP RAG mice.** Total protein from lobes of lungs from sham and CLP RAG mice was isolated via mechanical dispersion, and clarified via centrifugation. Cytokine levels in lung protein were analyzed via multiplex bead assay (Luminex), and protein levels were standardized by the total protein in each lung sample (obtained via Bradford protein assay). Data presented is representative of two separate experiments, with triplicate wells of samples from sham and CLP RAG mice, n = 5 mice per group. The limit of detection for each cytokine was routinely <5 pg/ml. (*) = p<0.05 vs. sham RAG, SEA-stimulated.
(TIFF)

Figure S4 **Chemokine mRNA expression in PPD- and SEA- challenged lungs.** Lobes of lungs from sham and CLP RAG mice four days following PPD- or SEA-bead challenge were isolated, and mRNA was isolated via phenol/chloroform extraction following mechanical dispersion. Expression of (A&D) CCL11, (B&E) CCL22 and (C&F) CXCL10 in (A–C) PPD- and (D–F) SEA-bead challenged mice was analyzed via quantitative real-time PCR with GAPDH expression used for standardization. Fold expression is displayed relative to expression levels in sham RAG lungs. Data presented is representative of two separate experiments, n = 5 mice per group. (*) = p<0.05 vs. sham RAG.
(TIFF)

Figure S5 **Schematic of the adoptive transfer and bead challenge model utilized in this study.**
(TIFF)

## References

[1] Perl TM, Dvorak L, Hwang T, Wenzel RP (1995). Long-term survival and function after suspected gram-negative sepsis.. JAMA.

[2] Quartin AA, Schein RM, Kett DH, Peduzzi PN (1997). Magnitude and duration of the effect of sepsis on survival. Department of Veterans Affairs Systemic Sepsis Cooperative Studies Group.. JAMA.

[3] Patenaude J, D'Elia M, Hamelin C, Bernier J (2010). Selective effect of burn injury on splenic CD11c(+) dendritic cells and CD8alpha(+)CD4(-)CD11c(+) dendritic cell subsets.. Cell Mol Life Sci.

[4] Duan X, Yarmush D, Leeder A, Yarmush ML, Mitchell RN (2008). Burn-induced immunosuppression: attenuated T cell signaling independent of IFN-gamma- and nitric oxide-mediated pathways.. J Leukoc Biol.

[5] Offner H, Vandenbark AA, Hurn PD (2009). Effect of experimental stroke on peripheral immunity: CNS ischemia induces profound immunosuppression.. Neuroscience.

[6] Cavassani KA, Carson Wt, Moreira AP, Wen H, Schaller MA (2010). The post sepsis-induced expansion and enhanced function of regulatory T cells creates an environment to potentiate tumor growth.. Blood.

[7] Benjamim CF, Hogaboam CM, Lukacs NW, Kunkel SL (2003). Septic mice are susceptible to pulmonary aspergillosis.. Am J Pathol.

[8] Deng JC, Cheng G, Newstead MW, Zeng X, Kobayashi K (2006). Sepsis-induced suppression of lung innate immunity is mediated by IRAK-M.. J Clin Invest.

[9] Wen H, Dou Y, Hogaboam CM, Kunkel SL (2008). Epigenetic regulation of dendritic cell-derived interleukin-12 facilitates immunosuppression after a severe innate immune response.. Blood.

[10] Cavaillon JM, Adib-Conquy M (2006). Bench-to-bedside review: endotoxin tolerance as a model of leukocyte reprogramming in sepsis.. Crit Care.

[11] Roth G, Moser B, Krenn C, Brunner M, Haisjackl M (2003). Susceptibility to programmed cell death in T-lymphocytes from septic patients: a mechanism for lymphopenia and Th2 predominance.. Biochem Biophys Res Commun.

[12] Napolitano LM, Campbell C (1995). Polymicrobial sepsis following trauma inhibits interleukin-10 secretion and lymphocyte proliferation.. J Trauma.

[13] Carson WFI, Cavassani KA, Ito T, Schaller M, Ishii M (2010). Impaired CD4+ T-cell proliferation and effector function correlates with repressive histone methylation events in a mouse model of severe sepsis.. Eur J Immunol.

[14] Zanotti-Cavazzoni SL, Goldfarb RD (2009). Animal models of sepsis.. Crit Care Clin.

[15] Benjamim CF, Lundy SK, Lukacs NW, Hogaboam CM, Kunkel SL (2005). Reversal of long-term sepsis-induced immunosuppression by dendritic cells.. Blood.

[16] Poehlmann H, Schefold JC, Zuckermann-Becker H, Volk HD, Meisel C (2009). Phenotype changes and impaired function of dendritic cell subsets in patients with sepsis: a prospective observational analysis.. Crit Care.

[17] Flohe SB, Agrawal H, Schmitz D, Gertz M, Flohe S (2006). Dendritic cells during polymicrobial sepsis rapidly mature but fail to initiate a protective Th1-type immune response.. J Leukoc Biol.

[18] Ding Y, Chung CS, Newton S, Chen Y, Carlton S (2004). Polymicrobial sepsis induces divergent effects on splenic and peritoneal dendritic cell function in mice.. Shock.

[19] Tinsley KW, Grayson MH, Swanson PE, Drewry AM, Chang KC (2003). Sepsis induces apoptosis and profound depletion of splenic interdigitating and follicular dendritic cells.. J Immunol.

[20] Ehlers S, Benini J, Held HD, Roeck C, Alber G (2001). Alphabeta T cell receptor-positive cells and interferon-gamma, but not inducible nitric oxide synthase, are critical for granuloma necrosis in a mouse model of mycobacteria-induced pulmonary immunopathology.. J Exp Med.

[21] Co DO, Hogan LH, Il-Kim S, Sandor M (2004). T cell contributions to the different phases of granuloma formation.. Immunol Lett.

[22] Chensue SW, Warmington K, Ruth J, Lincoln P, Kuo MC (1994). Cytokine responses during mycobacterial and schistosomal antigen-induced pulmonary granuloma formation. Production of Th1 and Th2 cytokines and relative contribution of tumor necrosis factor.. Am J Pathol.

[23] Ferguson NR, Galley HF, Webster NR (1999). T helper cell subset ratios in patients with severe sepsis.. Intensive Care Med.

[24] Ayala A, Chung CS, Xu YX, Evans TA, Redmond KM (1999). Increased inducible apoptosis in CD4+ T lymphocytes during polymicrobial sepsis is mediated by Fas ligand and not endotoxin.. Immunology.

[25] McDunn JE, Turnbull IR, Polpitiya AD, Tong A, MacMillan SK (2006). Splenic CD4+ T cells have a distinct transcriptional response six hours after the onset of sepsis.. J Am Coll Surg.

[26] Spolarics Z, Siddiqi M, Siegel JH, Garcia ZC, Stein DS (2003). Depressed interleukin-12-producing activity by monocytes correlates with adverse clinical course and a shift toward Th2-type lymphocyte pattern in severely injured male trauma patients.. Crit Care Med.

[27] Chensue SW, Warmington KS, Ruth JH, Lincoln P, Kunkel SL (1995). Cytokine function during mycobacterial and schistosomal antigen-induced pulmonary granuloma formation. Local and regional participation of IFN-gamma, IL-10, and TNF.. J Immunol.

[28] Chiu BC, Freeman CM, Stolberg VR, Komuniecki E, Lincoln PM (2003). Cytokine-chemokine networks in experimental mycobacterial and schistosomal pulmonary granuloma formation.. Am J Respir Cell Mol Biol.

[29] Wen H, Schaller MA, Dou Y, Hogaboam CM, Kunkel SL (2008). Dendritic cells at the interface of innate and acquired immunity: the role for epigenetic changes.. J Leukoc Biol.

[30] Murphey ED, Lin CY, McGuire RW, Toliver-Kinsky T, Herndon DN (2004). Diminished bacterial clearance is associated with decreased IL-12 and interferon-gamma production but a sustained proinflammatory response in a murine model of postseptic immunosuppression.. Shock.

[31] Ayala A, Deol ZK, Lehman DL, Herdon CD, Chaudry IH (1994). Polymicrobial sepsis but not low-dose endotoxin infusion causes decreased splenocyte IL-2/IFN-gamma release while increasing IL-4/IL-10 production.. J Surg Res.

[32] Ruth JH, Warmington KS, Shang X, Lincoln P, Evanoff H (2000). Interleukin 4 and 13 participation in mycobacterial (type-1) and schistosomal (type-2) antigen-elicited pulmonary granuloma formation: multiparameter analysis of cellular recruitment, chemokine expression and cytokine networks.. Cytokine.

[33] Khader SA, Bell GK, Pearl JE, Fountain JJ, Rangel-Moreno J (2007). IL-23 and IL-17 in the establishment of protective pulmonary CD4+ T cell responses after vaccination and during Mycobacterium tuberculosis challenge.. Nat Immunol.

[34] Okamoto Yoshida Y, Umemura M, Yahagi A, O'Brien RL, Ikuta K (2010). Essential role of IL-17A in the formation of a mycobacterial infection-induced granuloma in the lung.. J Immunol.

[35] Ayala A, Chung CS, Song GY, Chaudry IH (2001). IL-10 mediation of activation-induced TH1 cell apoptosis and lymphoid dysfunction in polymicrobial sepsis.. Cytokine.

[36] Joshi AD, Schaller MA, Lukacs NW, Kunkel SL, Hogaboam CM (2008). TLR3 modulates immunopathology during a Schistosoma mansoni egg-driven Th2 response in the lung.. Eur J Immunol.

[37] Smith PM, Shainheit MG, Bazzone LE, Rutitzky LI, Poltorak A (2009). Genetic control of severe egg-induced immunopathology and IL-17 production in murine schistosomiasis.. J Immunol.

[38] Hirata M, Kage M, Hara T, Yoneda Y, Zhang M (2001). Schistosoma japonicum egg granuloma formation in the interleukin-4 or interferon-gamma deficient host.. Parasite Immunol.

[39] Rezende SA, Oliveira VR, Silva AM, Alves JB, Goes AM (1997). Mice lacking the gamma interferon receptor have an impaired granulomatous reaction to Schistosoma mansoni infection.. Infect Immun.

[40] Cote-Sierra J, Foucras G, Guo L, Chiodetti L, Young HA (2004). Interleukin 2 plays a central role in Th2 differentiation.. Proc Natl Acad Sci U S A.

[41] Liao W, Schones DE, Oh J, Cui Y, Cui K (2008). Priming for T helper type 2 differentiation by interleukin 2-mediated induction of interleukin 4 receptor alpha-chain expression.. Nat Immunol.

[42] Laurence A, Tato CM, Davidson TS, Kanno Y, Chen Z (2007). Interleukin-2 signaling via STAT5 constrains T helper 17 cell generation.. Immunity.

[43] Bonecchi R, Bianchi G, Bordignon PP, D'Ambrosio D, Lang R (1998). Differential expression of chemokine receptors and chemotactic responsiveness of type 1 T helper cells (Th1s) and Th2s.. J Exp Med.

[44] Wen H, Hogaboam CM, Gauldie J, Kunkel SL (2006). Severe sepsis exacerbates cell-mediated immunity in the lung due to an altered dendritic cell cytokine profile.. Am J Pathol.

[45] Chensue SW, Warmington KS, Ruth JH, Sanghi PS, Lincoln P (1996). Role of monocyte chemoattractant protein-1 (MCP-1) in Th1 (mycobacterial) and Th2 (schistosomal) antigen-induced granuloma formation: relationship to local inflammation, Th cell expression, and IL-12 production.. J Immunol.

[46] Ito T, Schaller M, Hogaboam CM, Standiford TJ, Chensue SW (2007). TLR9 activation is a key event for the maintenance of a mycobacterial antigen-elicited pulmonary granulomatous response.. Eur J Immunol.

