# Supplementary figures and images for: Dysregulated Cytokine Expression by CD4+ T cells from Post-Septic Mice Modulates both Th1 and Th2-Mediated Granulomatous Lung Inflammation
Source: PLoS One. 2011 May 31;6(5):e20385. doi: 10.1371/journal.pone.0020385 (PMC3105020; doi:10.1371/journal.pone.0020385)

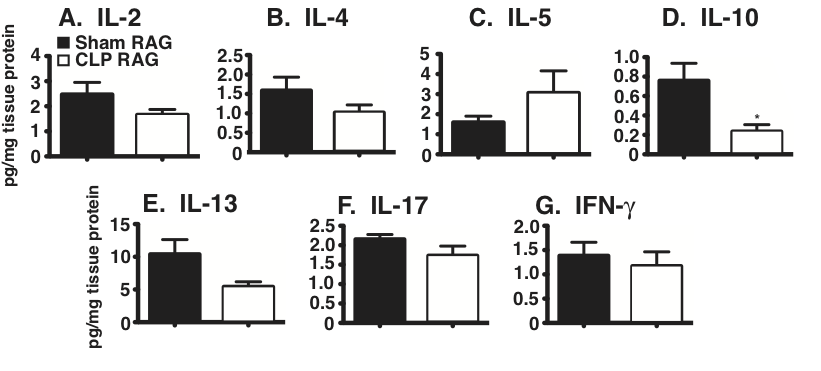

Supplement: Figure S1 — Cytokine expression in PPD-bead challenged lungs from sham and CLP RAG mice. Total protein from lobes of lungs from sham and CLP RAG mice was isolated via mechanical dispersion, and clarified via centrifugation. Cytokine levels in lung protein were analyzed via multiplex bead assay (Luminex), and protein levels were standardized by the total protein in each lung sample (obtained via Bradford protein assay). Data presented is representative of two separate experiments, with triplicate wells of samples from sham and CLP RAG mice, n = 5 mice per group. The limit of detection for each cytokine was routinely <5 pg/ml. (*) = p<0.05 vs. sham RAG, SEA-stimulated. (TIFF) [file pone.0020385.s001.tiff]

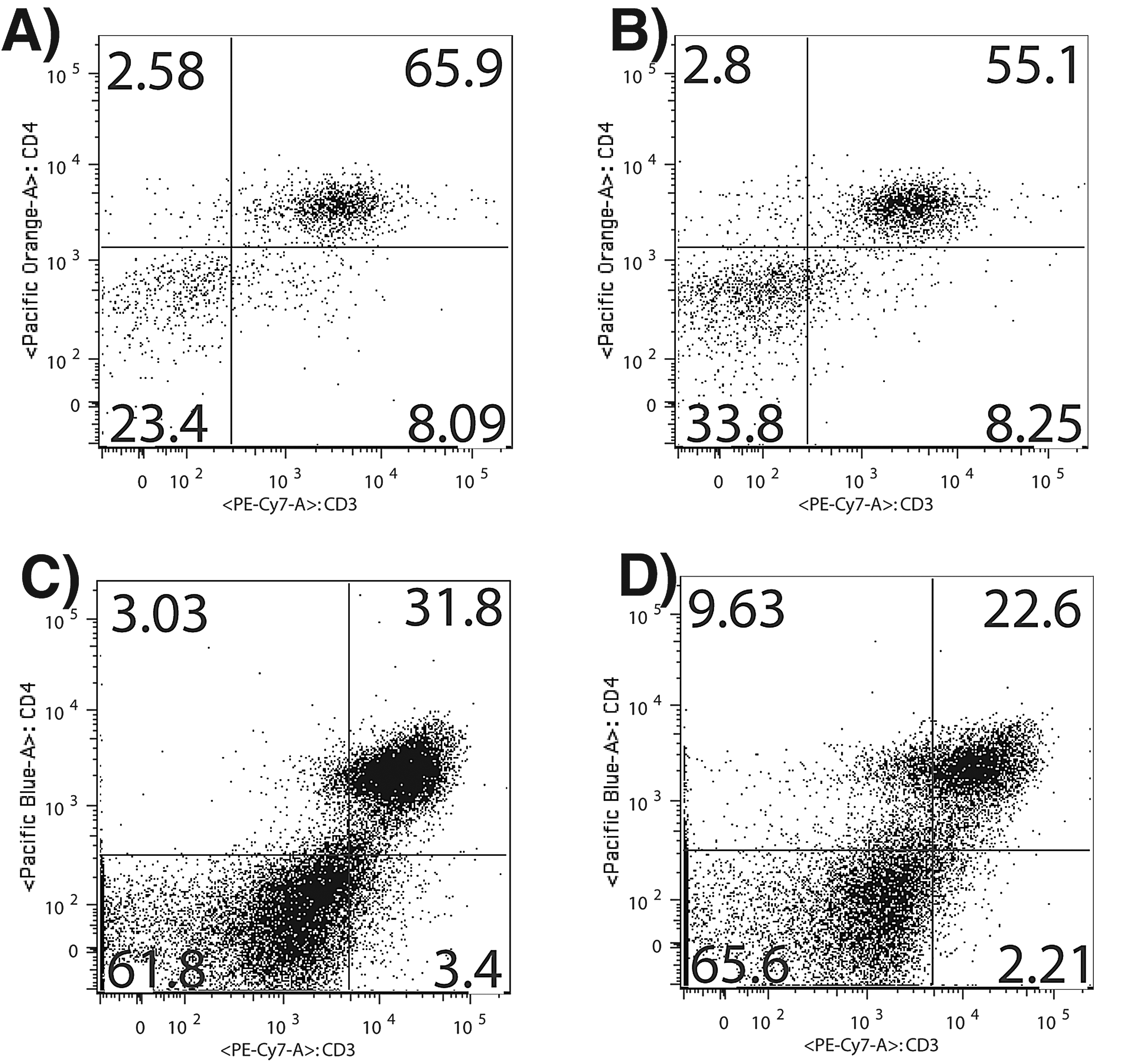

Supplement: Figure S2 — Flow cytometric analysis of lymph nodes from sham and CLP RAG mice. Individual lymph nodes from either sham (A&C) or CLP (B&D) PPD-challenged (A&B) or SEA-challenged (C&D) mice were processed individually and analyzed for the presence of CD3+ CD4+ T cells. Representative two-color plots were generated by gating on viable lymphocytes based on forward scatter (size) and side scatter (complexity) profiles, n = 4 mice per group. (TIFF) [file pone.0020385.s002.tiff]

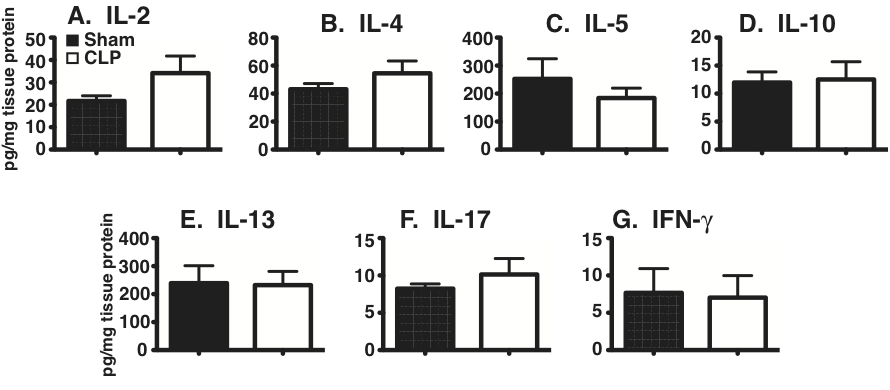

Supplement: Figure S3 — Cytokine expression in SEA-bead challenged lungs from sham and CLP RAG mice. Total protein from lobes of lungs from sham and CLP RAG mice was isolated via mechanical dispersion, and clarified via centrifugation. Cytokine levels in lung protein were analyzed via multiplex bead assay (Luminex), and protein levels were standardized by the total protein in each lung sample (obtained via Bradford protein assay). Data presented is representative of two separate experiments, with triplicate wells of samples from sham and CLP RAG mice, n = 5 mice per group. The limit of detection for each cytokine was routinely <5 pg/ml. (*) = p<0.05 vs. sham RAG, SEA-stimulated. (TIFF) [file pone.0020385.s003.tiff]

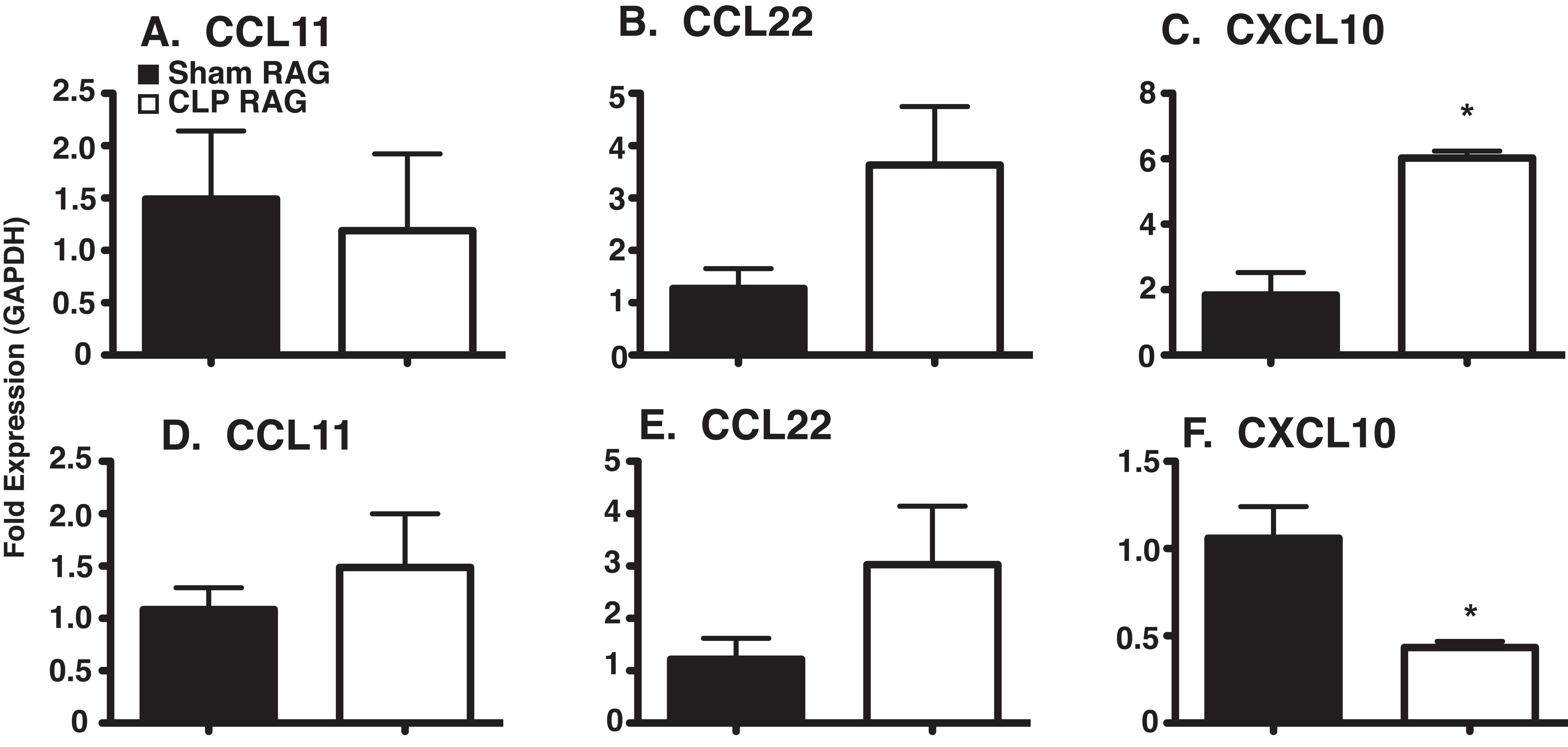

Supplement: Figure S4 — Chemokine mRNA expression in PPD- and SEA- challenged lungs. Lobes of lungs from sham and CLP RAG mice four days following PPD- or SEA-bead challenge were isolated, and mRNA was isolated via phenol/chloroform extraction following mechanical dispersion. Expression of (A&D) CCL11, (B&E) CCL22 and (C&F) CXCL10 in (A–C) PPD- and (D–F) SEA-bead challenged mice was analyzed via quantitative real-time PCR with GAPDH expression used for standardization. Fold expression is displayed relative to expression levels in sham RAG lungs. Data presented is representative of two separate experiments, n = 5 mice per group. (*) = p<0.05 vs. sham RAG. (TIFF) [file pone.0020385.s004.tiff]

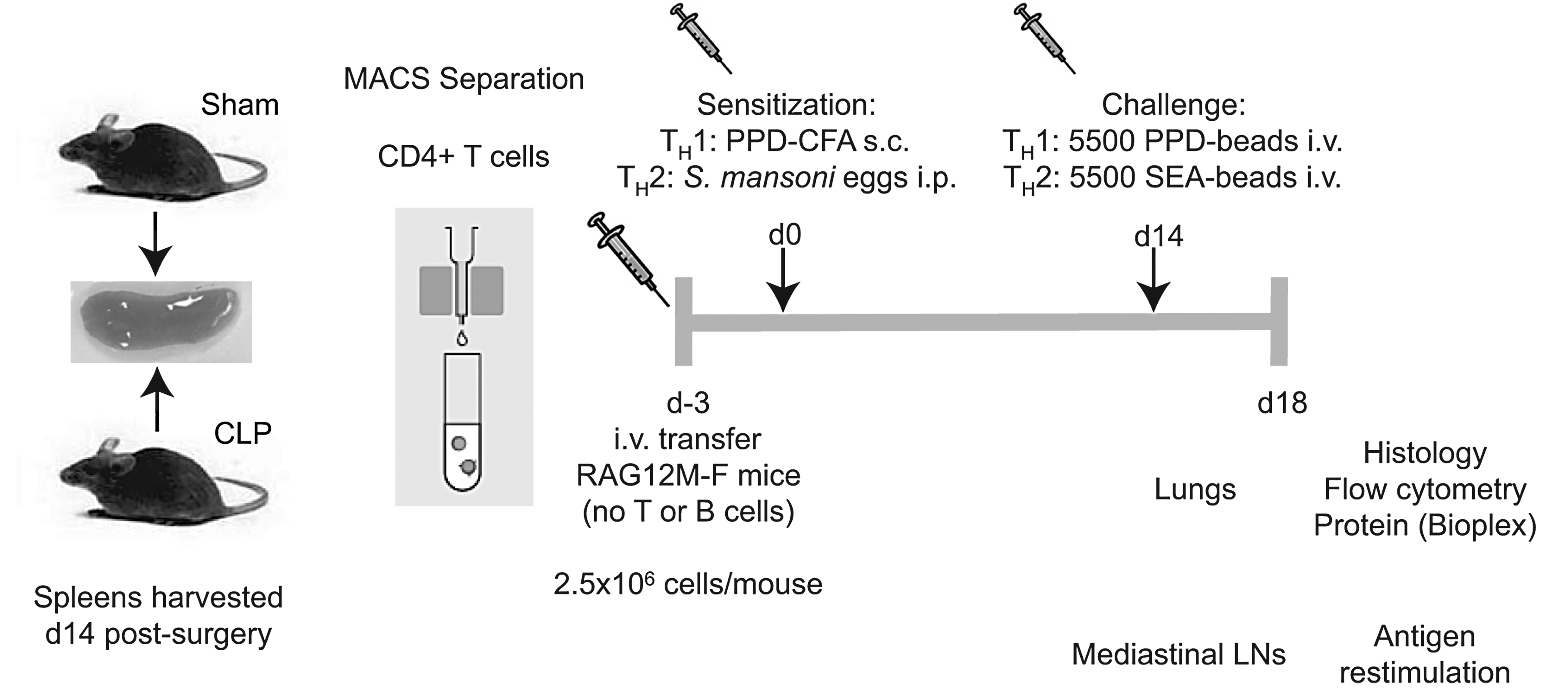

Supplement: Figure S5 — Schematic of the adoptive transfer and bead challenge model utilized in this study. (TIFF) [file pone.0020385.s005.tiff]
